# The quality of life index: a pilot study integrating treatment efficacy and quality of life in oncology

**DOI:** 10.1038/s41523-020-00193-6

**Published:** 2020-10-14

**Authors:** A. Basu, E. J. Philip, B. Dewitt, J. Hanmer, A. Chattopadhyay, C. Yau, M. E. Melisko, L. J. Esserman

**Affiliations:** 1 Department of Surgery, University of California San Francisco, San Francisco, CA 94115 USA; 2 School of Medicine, University of California San Francisco, San Francisco, CA 94115 USA; 3 Department of Engineering & Public Policy, Carnegie Mellon University, Pittsburgh, PA USA; 4 Division of General Internal Medicine, University of Pittsburgh, Pittsburgh, PA USA

**Keywords:** Signs and symptoms, Breast cancer

## Abstract

The majority of women diagnosed with breast cancer will experience some form of drug-related toxicity and subsequent impairments in Health-related Quality of Life (HRQoL). Despite this, HRQoL is assessed inconsistently and there is no validated method to integrate HRQoL data into the assessment of therapeutic agents. This proof of concept study utilizes data from the neoadjuvant I-SPY 2 clinical trial to describe the development of the Quality of Life Index (QoLI) measure. The QoLI represents a single composite score that incorporates validated longitudinal measures of clinical efficacy and QoL and one that permits a more comprehensive, direct comparison of individual therapeutic agents. Preliminary data suggest the QoLI is able to distinguish between agents based on their efficacy and toxicity; with further validation, the QoLI has the potential to provide more patient-centered evaluations in clinical trials and help guide treatment decision making in breast cancer and other oncologic diseases.

## Introduction

Breast cancer remains the most frequently diagnosed cancer among women worldwide, accounting for a quarter of all diagnoses. Despite advances in treatment and symptom management, the majority of women will experience some form of drug-related toxicity, psychosocial distress, and subsequent impairments in their Health-related Quality of Life (HRQoL), during the course of their illness^1–4^. Distress and impairments in HRQoL can interfere with treatment adherence, potentially decreasing survival^5^, while effective management of symptoms and engagement in health-promoting behaviors has been associated with improved HRQoL, adherence and increased survival^6,7^. Despite this growing evidence base, along with various efforts by governing bodies to integrate HRQoL data into the assessment of oncologic agents^8^, the utilization of HRQoL or other Patient-Reported Outcome (PRO) measures in clinical trials remains inconsistent. Further, while emerging technologies to collect and analyze patient-reported outcomes (PROs) have emerged, none thus far provide the ability to integrate measures of clinical efficacy and the impact of treatment on HRQoL. Indeed, there exist minimal standardized ways to: (1) assess whether patients are experiencing the same type and degree of HRQoL impairments across studies and treatments longitudinally and (2) formally integrate efficacy and toxicity as factors in assessing various therapeutic agents. New methods to analyze and visualize PRO data would add value to how toxicity reporting is managed in clinical trials today by providing a more comprehensive reporting of drugs and their accompanying side effects.

The collection of PROs and utilization of HRQoL, as standard practice in the clinical trial setting, would provide a more comprehensive, patient-centered assessment of therapies under development and ultimately help guide patient-provider discussions of treatment options in clinical care. The current study utilizes data from the I-SPY 2 TRIAL, an adaptive neoadjuvant breast cancer clinical trial platform designed to evaluate the benefit of new therapeutics, in combination with chemotherapy, to achieve complete disappearance of tumor by the time of surgery or a residual cancer burden (RCB) of zero. While tumor burden and HRQoL can be independently assessed, there is currently no valid or tested approach to integrate these two important aspects of drug assessment.

There were two goals of the current proof of concept pilot study: first, to demonstrate that it is feasible to report an integrated utility-based HRQoL score, the PROPr score, over the course of neoadjuvant therapy. As a measure of health utility, PROPr scores can be followed longitudinally to calculate Quality Adjusted Life Years (QALYs) experienced over the course of treatment. Second, to present a novel approach to calculating a single numerical index that integrates HRQoL and clinical efficacy (i.e. RCB) to generate a single composite score, the Quality of Life Index (QoLI). Once validated, the QoLI will provide a more comprehensive assessment of therapeutic agents in the context of randomized oncologic clinical trials and enable comparison of drugs within and across cancer types. Whereas severe adverse events are routinely reported, these provide no insight into the impact of such events on a patients’ HRQoL, or their effect over time. We anticipate that the integration of efficacy and toxicity will become routine in clinical trial assessment and will permit more fine-grained and patient-centered data to be generated to help guide patient care.

## Results

### HRQOL trajectory across arms

**Fig. 1:**
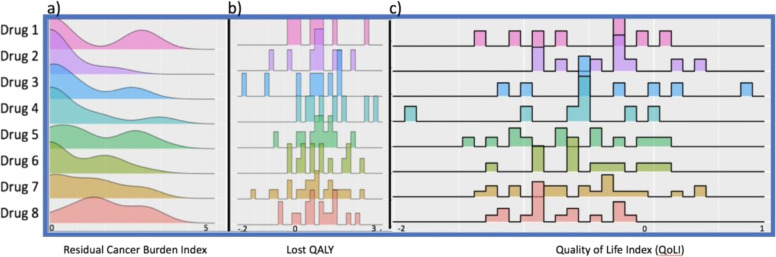
Distribution of Clinical Efficacy and Quality of Life Measures. Density plots of each therapeutic agent reflecting (**a**) RCB, (**b**) Lost QALY, and (**c**) QoLI.

Patients’ HRQoL trajectory was similar across each study arm, demonstrating impairment during the treatment period, and then gradual recovery in the month post-surgery (Supplementary Fig. 1). The Lost QALY index demonstrated a range of outcomes, with some arms clearly more challenging to tolerate, and others much better, with values ranging from −0.185 to 0.24 (Fig. 1). As expected, for the majority of patients, their baseline HRQoL was higher than their post-treatment HRQoL and thus recorded a decline in QALYs over the course of the study (Fig. 1). The RCB index of the seven study arms ranged from 0 to 4.35 (Fig. 1a).

### QOLI comparison across arms

The QoLI, an integration of validated assessments of clinical efficacy and HRQoL, can help garner insight into the patient experience and guide comparisons of therapeutic regiments. For example, Drug 3 and 4 both possessed similar distribution and mean values on the RCB index (0.87 vs. 0.85), suggesting similar clinical efficacy (Fig. 1a); however, examination of the Lost QALY and QoLI (ranging from −1.75 to 0.83) measures suggest that Drug 3 is less toxic and overall better tolerated by patients (Fig. 1b, c).

## Discussion

We are reporting the development of a novel, standardized assessment that could become a routine part of clinical trials in oncology. This proof of concept study, utilizing real-world clinical trial data from a neoadjuvant setting, suggests that the calculation of the QoLI is feasible and can reveal differences in the clinical profiles of therapeutic agents, both in terms of HRQoL and the integration of HRQoL and clinical efficacy. The current proof-of-concept study is not without limitations, with the major caveat of these analyses concerning sample size. The adaptive design of the I-SPY 2 TRIAL efficiently and rapidly identifies agent-subtype combinations based on their estimated likelihood of phase III success; as a consequence of this study design, relatively few patients may be recruited to certain study arms. Moreover, the number of patients with completed questionnaires across all three timepoints further limited the current analysis. Finally, responses were not collected beyond the 1-month post-surgery timepoint and thus the long-term toxicity of agents is unknown. Future studies are planned to address this issue and will generate QALY information for up to 24 months post treatment. The collection of such data may also help motivate more timely interventions to abrogate side effects in cancer care.

The QoLI represents a novel approach to providing summary data that can be easily interpreted as part of neoadjuvant clinical trial outcome data. Ideally, these integrated assessments can provide a more comprehensive evaluation of investigational therapies, and ultimately help inform treatment decisions and discussions between patients and providers. Further, once validated, the QoLI index could be employed successfully in other disease domains and settings, including adjuvant, survivorship, and metastatic disease, providing there was an objective measure of treatment response and the utilization of a HRQoL measure that permits calculation of QALYs. Further, although we have presented data in this pilot study as such, additional methods and metrics could be used to present such holistic data moving forward. For example, mean values could be plotted by the treatment arm and utilized in the evaluation of agents, as well as help guide discussions between patients and providers regarding the risks and benefits associated with a particular agent. Should two agents possess a similar efficacy profile, as noted in the study example, then their associated toxicity can be used as a defining feature in their evaluation.

Moving forward, electronic PRO data should be collected as part of routine care in clinical trials, thus enabling longitudinal HRQoL and QoLI scores to be generated for every agent evaluated. This real-world data would help guide discussions among providers and patients, as well as inform regulators and pharmaceutical companies concerning the efficacy and toxicity of new agents once they are introduced to the market.

## Methods

### Cohort characteristics

551 patients completed baseline paper-based surveys; of these, 18.5% (*n* = 102) had complete data across all three study time-points. Our analysis focuses on the 102 patients with complete data rather than conducting imputation, and thus our data represent a proof of concept study. Study participants were part of the I-SPY 2 TRIAL that assesses novel neoadjuvant therapies added to standard chemotherapy in the treatment of Stage 2/3 breast cancer. Patients in the study were randomized to the control arm or experimental drug arms, with patients in the control arm treated with Paclitaxel followed by anthracycline (AC). Participants completed a validated HRQoL measure at three time points: baseline, prior to surgery, and 1-month post-surgery. HRQoL was assessed using the NIH Patient-Reported Outcomes Measurement Information System (PROMIS®) measure and results at each time-point were used to calculate the PROPr score, a single utility-based index score to assess overall HRQoL. PROPr is a preference-based summary score of HRQoL that is constructed from seven PROMIS domains: cognitive function, depression, fatigue, pain interference, physical function, sleep disturbance, and ability to participate in social roles and activities^9,10^. The PROPr scoring algorithm was developed from standard gamble valuations of a US-based sample^11^ and scores range from −0.022 to 1.0. The current study collected 5 of the 7 necessary domains to calculate PROPr; the remaining 2 (Pain Interference and Sleep Disturbance) were simulated in order to calculate PROPr. While the estimation of missing domains, as employed in the current study, have been shown to be an acceptable strategy in previous research^12^, future studies will contain all seven domains.

### Calculation of QALY

**Fig. 2:**
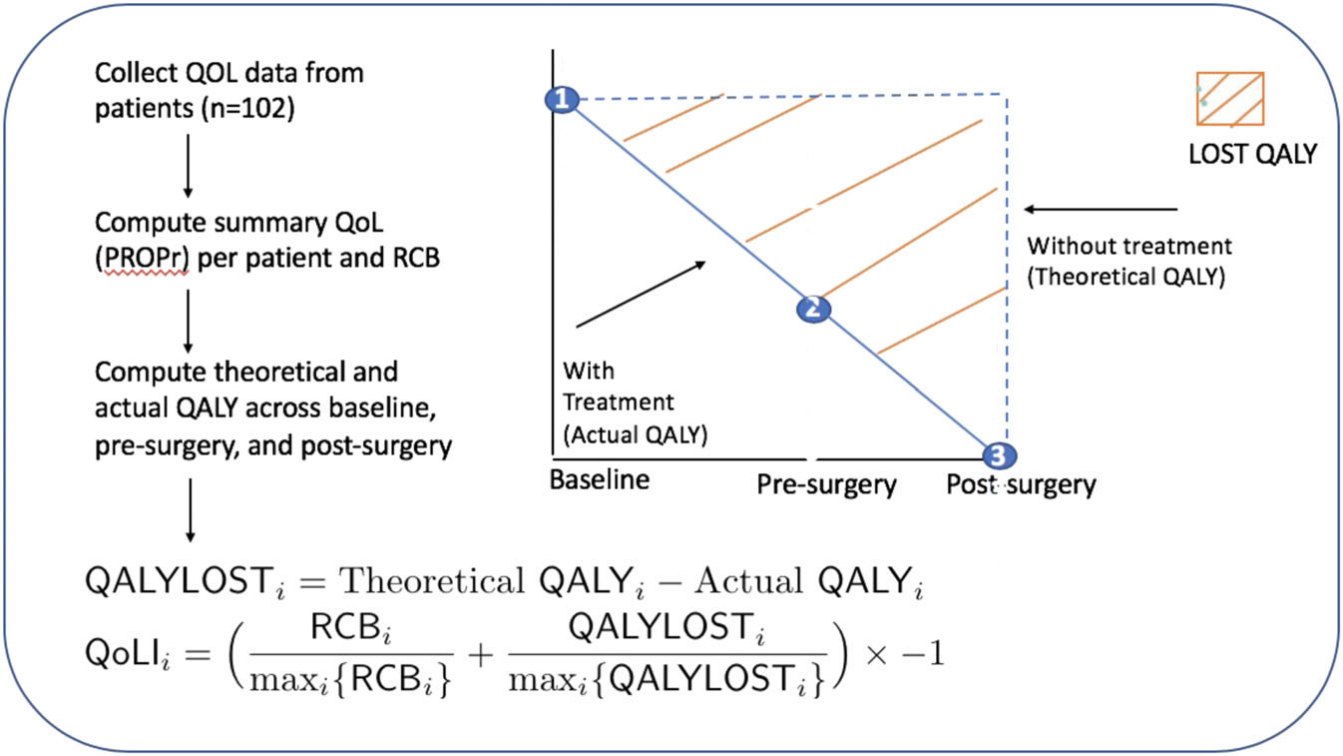
Quality of life data is collected from patients at each time-point and used to derive QALYs without treatment and actual QALYs for each patient. The difference between these two represents HRQoL lost during treatment and when combined with their residual cancer burden, provides the QoL-adjusted Clinical Efficacy (QoLI) index.

In the current pilot study, the PROPr utility score was calculated at three time points to generate an estimate of the QALYs experienced by the patient during the course of treatment^13,14^. Patients’ actual QALY was calculated by estimating the PROPr score at each measurement time-point, calculating the QALYs experienced during the intervening time period, and then summing the QALYs across treatment (baseline->pre-surgery + pre-surgery->post-surgery) (Fig. 2). To calculate the theoretical QALY, we assumed that the baseline HRQOL would remain constant over a 6-month period of time in the absence of treatment. The number of actual QALYs was then deducted from the theoretical QALYs reflecting the lost QALYs over the course of treatment, with higher numbers representing greater treatment burden and impairment. Clinical efficacy was based on the RCB observed at the time of surgery. RCB is expected to vary from patient to patient, depending on their response to neoadjuvant therapy. An RCB of 0 reflects the achievement of a pathological complete response, with no residual invasive disease in breast and nodes, and increases with increasing amount of residual disease. 4.35 was the maximum RCB among patients in this study.

### Calculation of QoLI

The QoLI is generated by (1) dividing the RCB Index over the maximum RCB index (4.35), and dividing the Lost QALY over the maximum Lost QALY (equal to 0.54 with PROPr = 1), (2) summing the two proportions, and (3) reversing the direction of the sum by multiplying by −1 so that a higher QoLI is indicative of a higher HRQOL-adjusted clinical efficacy. Notably, a patient can also gain QALY, and a negative Lost QALY reflects this measure. Hence, a higher QoLI represents agents that possess the greatest clinical efficacy with the least amount of burden on patients’ HRQoL (Fig. 2). An example calculation of the QoLI is provided in the Supplementary Material. For example, assuming a person with a RCB = 2 had the same Lost QALY as a person with a RCB = 0.02, the person with a lower RCB index (less residual cancer) should have a higher QoLI (Supplemental Table 1).

The research study presented in this paper has complied with ethical standards and received ethical approval from the Institutional Review Board (#10-01565) at the relevant participating institutions, and all participants were treated in accordance with the Ethical Standards of the American Medical Association. All participants in this study provided written informed consent to take part in the study.

### Reporting summary

Further information on research design is available in the Nature Research Reporting Summary linked to this article.

## Supplementary information

Reporting Summary

Supplemental Information

## Data Availability

The data generated and analyzed during this study are described in the following data record: 10.6084/m9.figshare.12765227^15^. The data underlying Fig. 1 and Supplementary Figure were acquired from patients via the I-SPY 2 clinical trial. These data cannot be made directly available, but interested parties can apply to access the I-SPY data by submitting the standard I-SPY 2 Concept Proposal Form following the process described on the I-SPY Trials’ Proposal Submissions page here: https://www.ispytrials.org/collaborate/proposal-submissions. The data underlying Supplementary Table 1 is available as part of this metadata record (SuppTable1.pdf), and is also available in GitHub here: http://github.com/amritabasu1977/ISPYQOL.
