# Supplementary material for: The quality of life index: a pilot study integrating treatment efficacy and quality of life in oncology
Source: NPJ Breast Cancer. 2020 Oct 14;6:52. doi: 10.1038/s41523-020-00193-6 (PMC7560724; doi:10.1038/s41523-020-00193-6)
Supplement: Supplementary file 1 — Reporting Summary [file 41523_2020_193_MOESM1_ESM.pdf]

nature research

Corresponding author(s): Amrita Basu

Last updated by author(s): Jul 1, 2020

Reporting Summary

Nature Research wishes to improve the reproducibility of the work that we publish. This form provides structure for consistency and transparency in reporting. For further information on Nature Research policies, see our Editorial Policies and the Editorial Policy Checklist.

Statistics

For all statistical analyses, confirm that the following items are present in the figure legend, table legend, main text, or Methods section.

n/a	Confirmed	
☐	☒	The exact sample size (n) for each experimental group/condition, given as a discrete number and unit of measurement
☒	☐	A statement on whether measurements were taken from distinct samples or whether the same sample was measured repeatedly
☒	☐	The statistical test(s) used AND whether they are one- or two-sided<br>*Only common tests should be described solely by name; describe more complex techniques in the Methods section.*
☐	☒	A description of all covariates tested
☐	☒	A description of any assumptions or corrections, such as tests of normality and adjustment for multiple comparisons
☐	☒	A full description of the statistical parameters including central tendency (e.g. means) or other basic estimates (e.g. regression coefficient) AND variation (e.g. standard deviation) or associated estimates of uncertainty (e.g. confidence intervals)
☒	☐	For null hypothesis testing, the test statistic (e.g. F, t, r) with confidence intervals, effect sizes, degrees of freedom and P value noted<br>*Give P values as exact values whenever suitable.*
☒	☐	For Bayesian analysis, information on the choice of priors and Markov chain Monte Carlo settings
☐	☒	For hierarchical and complex designs, identification of the appropriate level for tests and full reporting of outcomes
☒	☐	Estimates of effect sizes (e.g. Cohen's d, Pearson's r), indicating how they were calculated

Our web collection on statistics for biologists contains articles on many of the points above.

Software and code

Policy information about availability of computer code

Data collection	All code was written in R and will be available through github (https://github.com/amritabasu1977/ISPYQOL)
Data analysis	We used R Version 1.1.463 for the Macintosh.

For manuscripts utilizing custom algorithms or software that are central to the research but not yet described in published literature, software must be made available to editors and reviewers. We strongly encourage code deposition in a community repository (e.g. GitHub). See the Nature Research guidelines for submitting code & software for further information.

Data

Policy information about availability of data

All manuscripts must include a data availability statement. This statement should provide the following information, where applicable:

- Accession codes, unique identifiers, or web links for publicly available datasets
- A list of figures that have associated raw data
- A description of any restrictions on data availability

The data supporting results and figures in this published article are not publicly available but may be accessible from the corresponding author upon request.

Field-specific reporting

Please select the one below that is the best fit for your research. If you are not sure, read the appropriate sections before making your selection.

☐ Life sciences ☒ Behavioural & social sciences ☐ Ecological, evolutionary & environmental sciences

For a reference copy of the document with all sections, see nature.com/documents/nr-reporting-summary-flat.pdf

Behavioural & social sciences study design

All studies must disclose on these points even when the disclosure is negative.

Study description	We collected survey data from patients (qualitative) and then analyzed the data using quantitative methods.
Research sample	551 I-SPY breast cancer clinical trial patients, 102 of these patients had complete data for 3 study timepoints.
Sampling strategy	All patients that had completed at least 3 study timepoints.
Data collection	We used PROMIS survey instrument to collect 5 domains of data.
Timing	Baseline, Pre-surgery (1 month), Post surgery (1 month)
Data exclusions	Exclusion criteria include those patients that did not complete surveys at the 3 timepoints
Non-participation	No participants dropped out.
Randomization	Patients were already randomized to either the experimental arm or control arm as per the I-SPY design.

Reporting for specific materials, systems and methods

We require information from authors about some types of materials, experimental systems and methods used in many studies. Here, indicate whether each material, system or method listed is relevant to your study. If you are not sure if a list item applies to your research, read the appropriate section before selecting a response.

Materials & experimental systems

n/a	Involved in the study	
☒	☐	Antibodies
☒	☐	Eukaryotic cell lines
☒	☐	Palaeontology and archaeology
☒	☐	Animals and other organisms
☐	☒	Human research participants
☐	☒	Clinical data
☒	☐	Dual use research of concern

Methods

n/a	Involved in the study	
☒	☐	ChIP-seq
☒	☐	Flow cytometry
☒	☐	MRI-based neuroimaging

Human research participants

Policy information about studies involving human research participants

Population characteristics	See above
Recruitment	Patients that filled out consent to be part of the study.
Ethics oversight	IRB

Note that full information on the approval of the study protocol must also be provided in the manuscript.

Clinical data

Policy information about clinical studies

All manuscripts should comply with the ICMJE guidelines for publication of clinical research and a completed CONSORT checklist must be included with all submissions.

Clinical trial registration	NCT01042379

Study protocol	*Note where the full trial protocol can be accessed OR if not available, explain why.*
Data collection	26 study locations in the US, all active and most recruiting
Outcomes	Determine whether adding experimental agents to standard neoadjuvant medications increases the probability of pathologic complete response (pCR) over standard neoadjuvant chemotherapy for each biomarker signature established at trial entry.
