# Supplementary material for: The quality of life index: a pilot study integrating treatment efficacy and quality of life in oncology
Source: NPJ Breast Cancer. 2020 Oct 14;6:52. doi: 10.1038/s41523-020-00193-6 (PMC7560724; doi:10.1038/s41523-020-00193-6)
Supplement: Supplementary file 2 — Supplemental Information [file 41523_2020_193_MOESM2_ESM.pdf]

Supplementary Figure 1. Mean participants' quality of life (PROPr) scores per therapeutic agent as measured at baseline, pre-surgery and 1-month post-surgery.

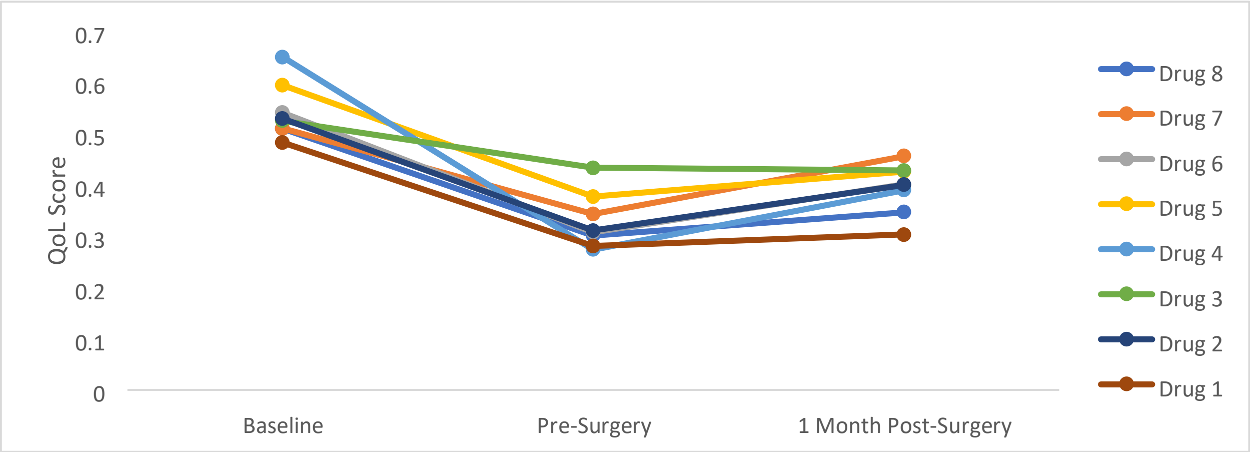

Supplementary Table 1. Example calculation of the QoLI

	**HRQoL (PROPr) Score**	**RCB**	**Max. RCB**	**Max. LOST QALY**
**Baseline**	0.5			
**Pre-Surgery**	0.4	2	4.35	.54
**Post-Surgery**	0.3			
**QALY if no treatment and QoL maintained**	0.5 x 6.5/12* = 0.27 QALYs			
**QALY from baseline to pre-surgery (5.5 months)**	**Avg HRQoL x 5.5/12*** (0.5+0.4)/2 x 5.5/12 = 0.20 QALYs			
**QALY from pre-surgery to post-surgery (1 month)**	**Avg HRQoL x 1/12*** (0.4+0.3)/2 x 1/12 = 0.05 QALYs			
**Total QALYs Lost During Treatment**	0.27 – (0.20+0.05) = 0.02 QALYs. Thus, had they maintained baseline they would have enjoyed .02 QALYs more, which equates to 7.3 days at full health.			
**Lost QALY**	0.02			
**QoLI**	(2/4.35) + (.02/.54)= (.46 +.04) * -1 =-0.5			

*Division by 12 converts the timeframe to one year and thus enables QALYs to be calculated

Assuming LOST HRQOL = .02 (as in table above)

RCB = .02 ; QoLI= .02/4.35 + **+.02/.54 = .004 + .040 = .044* -1 = -.044**

RCB = .01 ; **QoLI** = .01/4.35 + **+.02/.54 = .003 + .040 = .043 * -1 = -.043 -> higher QoLI than line above**
